# Intervention for adolescent tobacco initiation prevention (IATIP) to promote health and well-being: a protocol for a pilot cluster randomised controlled trial in Dhaka, Bangladesh

**DOI:** 10.1186/s40814-025-01710-9

**Published:** 2025-11-17

**Authors:** Sahadat Hossain, Emma Beard, Lion Shahab

**Affiliations:** 1 Department of Behavioural Science and Health, Institute of Epidemiology and Health Care, University College London, London, UK; 2 Department of Public Health and Informatics, Jahangirnagar University, Savar, Dhaka, Bangladesh; 3 SPECTRUM Consortium, Edinburgh, UK

**Keywords:** Adolescent, Tobacco initiation, Health and well-being, Prevention, School-based intervention, Bangladesh

## Abstract

**Background:**

Adolescent tobacco use remains a significant public health concern in low- and middle-income countries (LMICs) such as Bangladesh. To address this issue, an Intervention for Adolescent Tobacco Initiation Prevention (IATIP) has been developed. This study aims to assess the feasibility of implementing the IATIP and to evaluate its potential efficacy in preventing tobacco initiation and promoting adolescent health and well-being.

**Methods:**

This pilot school-based cluster randomised controlled trial will be conducted among adolescents (*N *≈ 1280 participants) in school years 8 and 9 attending participating schools (*K* = 8 schools) in Dhaka, Bangladesh. The intervention consists of three 40-min sessions delivered over three successive days, incorporating knowledge- and skill-based lectures, interactive activities, and materials targeting tobacco prevention and health promotion. The intervention was developed based on evidence from a systematic review, a quantitative survey, qualitative focus groups, and expert consensus. The primary outcome is intervention feasibility, assessed through recruitment and attrition rates, fidelity of delivery, and acceptability. Secondary outcomes include changes in tobacco use intentions, tobacco-related knowledge, attitudes and beliefs, skills development, and measures of health and well-being. Data will be collected through structured questionnaires at baseline and at 1-month follow-up. A post-intervention process evaluation will be conducted with a sub-sample of pupils in the intervention arm and their teachers, using a mixed-methods approach to explore implementation processes, facilitation, participant experiences, and areas for improvement.

**Discussion:**

This pilot trial addresses a critical gap in adolescent tobacco control in LMICs. By combining evidence-based strategies, the IATIP aims to equip adolescents with the knowledge and skills necessary to resist tobacco initiation and to enhance overall health and well-being. Successful implementation of the intervention may contribute to reduced tobacco initiation, lower exposure to second-hand smoke, and improved adolescent health outcomes. Given the scalability of school-based interventions, the findings of this study may inform future large-scale definitive trials and influence national policies and strategies.

**Trial registration:**

ClinicalTrials.gov (NCT06399588)

**Supplementary Information:**

The online version contains supplementary material available at 10.1186/s40814-025-01710-9.

## Background

Tobacco use remains a major global public health challenge, contributing substantially to both mortality and morbidity [1, 2]. In 2019, tobacco-related causes were responsible for 8.7 million deaths (15.4% of all deaths) and 229.8 million disability-adjusted life years lost worldwide, with the burden particularly pronounced in low- and middle-income countries (LMICs) [3]. Beyond its well-documented physical health consequences, tobacco use is intricately linked to mental health through a bidirectional relationship: tobacco use can contribute to mental health disorders such as anxiety, depression, and substance use disorders [4–8], while individuals with pre-existing mental health conditions are more likely to use tobacco as a form of self-medication or coping mechanism [5, 6]. This, in turn, can exacerbate their symptoms and perpetuate a cycle of dependency [5, 6, 8]. Acknowledging the interplay between tobacco use and both physical and mental health disorders underscores the importance of adopting holistic preventive interventions [5].

Preventing tobacco initiation, particularly among adolescents, is a critical step in addressing the associated harms. Studies consistently indicate that the majority of tobacco users begin the habit before reaching the age of 15 [9, 10]. Tobacco use during adolescence is especially concerning due to its highly addictive nature [9, 11–13]. According to the World Health Organization, approximately 38 million adolescents worldwide—comprising 25 million boys and 13 million girls aged 13 to 15—currently use tobacco [14]. Preventing tobacco use during this developmental stage can significantly enhance adolescents’ overall health and well-being. Health and well-being encompass various dimensions, including physical health, psychological resilience, and social engagement [15]. Physical wellness involves self-care and the adoption of a balanced lifestyle, while psychological well-being includes subjective experiences and the presence of positive emotions [16]. A stable emotional state enhances resilience, and social engagement fosters a sense of belonging and support through interpersonal relationships [17]. These facets are integral to adolescent health interventions, including those aimed at preventing tobacco use.

Given that adolescents spend a considerable portion of their time in schools, educational settings represent a key platform for promoting adolescent health and well-being [18, 19]. Schools have the capacity to reach the vast majority of adolescents, including those who may not regularly engage with health services [20]. Global evidence demonstrates that school-based interventions can be effective in preventing tobacco initiation through various approaches such as classroom lessons, anti-tobacco messaging, and peer communication [21–23].

Bangladesh, a densely populated LMIC, ranks among the top ten countries globally for tobacco use, encompassing both combustible and non-combustible products [24, 25]. With approximately 36 million adolescents constituting 22.0% of the population in the country, the situation demands attention [26]. According to the Global School-based Student Health Survey (2014), 9.8% of school-going adolescents in Bangladesh use tobacco, with a gender disparity of 13.8% among boys and 2.0% among girls [27]. Additionally, 31.1% of adolescents are exposed to tobacco smoke at home, and 59.0% are exposed in enclosed public places [28]. Multiple factors contribute to adolescent tobacco initiation, including familial and peer influences, low socioeconomic status, impulsivity, and mental health disorders [29]. Notably, the prevalence of common mental disorders among Bangladeshi adolescents is estimated to range between 25 and 37%, highlighting the need for targeted interventions [26, 30]. However, Bangladesh faces several challenges, including the ubiquitous availability of tobacco products, weak enforcement of existing regulations, and the absence of context-specific preventive interventions [24, 25, 31]. At present, there is no intervention programme in Bangladesh specifically designed to prevent tobacco initiation among adolescents while simultaneously promoting health and well-being.

The Intervention for Adolescent Tobacco Initiation Prevention (IATIP) has been developed to address this gap by providing insights and strategies tailored to the needs of Bangladeshi adolescents. Given the absence of similar interventions in Bangladesh and the context-specific challenges of school-based implementation, several feasibility uncertainties need to be addressed before designing a definitive trial. These include uncertainties around recruitment and retention of schools and students, acceptability of the intervention to adolescents and teachers, fidelity of intervention delivery in a real-world school setting, appropriateness of outcome measures, and the feasibility of follow-up assessments. The present study is designed to explore these key feasibility parameters, with the aim of determining whether a future large-scale randomised controlled trial would be practicable and, if so, how it might best be designed.

This protocol outlines a pilot trial to assess the feasibility and potential efficacy of the IATIP intervention, which will be implemented among adolescents (aged 11–17) in school years 8 and 9 in Dhaka, Bangladesh. This age group has been selected due to its heightened susceptibility to tobacco initiation and the critical importance of early intervention in preventing tobacco use [9, 10, 32]. While the primary aim of the intervention is to prevent tobacco initiation, it incorporates components that address aspects of health and well-being. Efforts to prevent tobacco use can indirectly promote physical, psychological, and social well-being, and vice versa, even if these are not the principal focus of the intervention [15–17]. This manuscript outlines the objectives, design, and methodology of the IATIP trial, contributing to the evidence base for adolescent tobacco prevention and broader adolescent health promotion in the context of Bangladesh.

### Trial objectives

#### Primary objective

To assess the feasibility of implementing the IATIP in the school setting among adolescents in Dhaka, Bangladesh.

#### Secondary objectives

1. To evaluate the potential efficacy of IATIP in preventing tobacco initiation as indexed by intention to use among school adolescents.
2. To examine the effect of IATIP on promoting health and well-being in the adolescents.
3. To explore changes in tobacco-related knowledge, attitudes, beliefs, and skills to resist tobacco.
4. To conduct a process evaluation to understand the implementation, facilitation, and participant experiences of the IATIP.

## Methods

### Trial design

This study employs a pilot cluster randomised controlled trial (C-RCT) design. The unit of randomization will be schools, with the ultimate aim of informing a full-scale C-RCT. In addition, a mixed-methods approach will be employed for the process evaluation.

### Setting

This study will be conducted in Dhaka, Bangladesh. Dhaka was selected as the setting due to its status as the capital and most densely populated urban centre in the country, with a high concentration of secondary schools and adolescents. The city represents a diverse population with varying socioeconomic backgrounds and a relatively high burden of adolescent tobacco use, making it a strategic location for piloting school-based prevention interventions. Furthermore, conducting the study in Dhaka allows for logistical feasibility, including access to research infrastructure and existing collaborations with local schools and education authorities.

### Study participants and eligibility

The trial will recruit students enrolled in years 8 and 9 in schools across Dhaka. Eligible participants must meet the following criteria: (i) be Bangladeshi school-going adolescents; (ii) be enrolled at one of the participating schools; and (iii) be in year 8 or 9. Exclusion criteria include (i) students unwilling to participate; (ii) students unable to provide informed consent (including parental consent); and (iii) students with severe cognitive or physical impairments that hinder participation or completion of assessments (as determined in consultation with school teachers).

### Intervention

This pilot trial introduces the IATIP, an evidence-based and culturally sensitive tobacco prevention programme designed to promote health and well-being specifically for Bangladeshi adolescents. The IATIP comprises three 40-min sessions delivered over three consecutive days. A detailed overview of the IATIP sessions—including session titles, subtopics, interactive components, learning outcomes, and learning methods—is presented in Table 1.

**Table 1:** Description of the IATIP sessions, including their title, sub-sessions’ taught and interactive components, learning outcomes, and learning types

Session	Title	Sub-session no.	Taught components	Interactive components	Learning outcomes	Learning types
1	Tobacco Talk: Understanding the Basics, Health Effects, and Perspectives	1.1	Tobacco and tobacco products	–	Students will acquire a basic understanding of the nature of tobacco, its compositions, and the various forms of tobacco products	Acquisition
		1.2	Tobacco toxicity and addiction	Word cloud game (group activity)	Students will gain insight into the addictive nature of tobacco and the physiological and psychological mechanisms that lead to addiction	Investigation Collaboration Acquisition
		1.3	Health effects of tobacco use	Word cloud game (group activity), video presentation, and artwork (poster contest)	Students will explore the detrimental health effects of tobacco use, including both short-term and long-term consequences	Investigation Collaboration Discussion Acquisition
		1.4	Religious views on Tobacco use	–	Students will learn how different religion’s view tobacco use and its implications on personal beliefs and values	Acquisition
2	Kick the Habit: Smart Decision and Strong Skills for a Healthy Lifestyle	2.1	Why do people use tobacco?	–	Students will gain the ability to identify and critically analyze factors influencing tobacco use behaviour	Acquisition
		2.2	Decision making and problem-solving skills to resist tobacco: assertiveness and refusal skills	Situational role playing to demonstrate skills (individual and group),	Students will be better equipped to make informed decisions regarding saying NO TO TOBACCO by developing essential life skills, including assertiveness and refusal skills	Acquisition Practice
		2.3	Discover positive and healthy alternatives to using tobacco	Group discussion word cloud game (group activity)	Students will explore and identify positive and healthy alternatives to tobacco use	Discussion Collaboration Production
3	Mind Matters: Navigating Emotions and Setting Goals for a Tobacco-Free Future	3.1	Links between tobacco and mental wellbeing	–	Students will gain an understanding of what mental well-being is, its significance, and how it relates to tobacco use prevention	Acquisition
		3.2	Coping with emotions: expression of negative feelings, dealing with stress, depression and anxiety	Deep breathing exercise	Students will develop skills to build emotional resilience, cope with stress, depression and anxiety, and manage emotions without turning to tobacco use	Acquisition Practice
		3.3	Goal setting and personal commitment (pledge) not to use tobacco	Open discussion Written pledges, and artwork (poster contest)	Students will be able to set clear and achievable goals related to tobacco use behaviour, whether it is becoming a tobacco non-user or achieving tobacco cessation. And also, students will reinforce their commitment to a tobacco-free lifestyle and how it will contribute to their well-being	Discussion Investigation Production

The development process of the IATIP consisted of four stages: (i) review of existing literature, (ii) needs assessment, (iii) selection of intervention components and framework integration, and (iv) expert consensus and adaptation. In the first stage, a comprehensive review of existing literature on adolescent tobacco use prevention strategies was performed [21]. This literature review aimed to explore the effectiveness of school-based tobacco prevention interventions in LMICs and identify key risk factors, protective factors, and evidence-based interventions relevant to the Bangladeshi context. Following the literature review, a needs assessment was conducted to better understand the specific challenges faced by adolescents in Bangladesh regarding tobacco use and health and well-being. This assessment included a large-scale school survey and a qualitative study—focus groups and interviews with students, teachers, and parents—to gather insights into the social, cultural, and environmental factors influencing adolescent behaviour. In the third stage, the intervention components were selected based on the findings of stages 1 and 2. Rooted in evidence-based practices, the components were integrated with Behaviour Change Techniques [33], the Behaviour Change Wheel [34], and the Theoretical Domains Framework [35]. To ensure the delivery of the intervention is both effective and engaging, the Arena Blended Connected (ABC) model was applied [36]. This model combines a variety of delivery methods, including face-to-face sessions, online resources, and interactive technologies, to create a dynamic and accessible learning experience for adolescents. The diverse learning needs of adolescents were acknowledged, and six key learning types were integrated to optimise knowledge acquisition, concept investigation, collaboration, meaningful discussions, practical application, and active skill and insight production. In the fourth stage, an expert consensus workshop similar to a Delphi exercise was performed with nine international experts in the fields of tobacco control, psychology, behaviour change, public health, and education. The draft version of the intervention contents underwent thorough review by the experts to ensure its scientific rigour and alignment with evidence-based practices. Based on the feedback of the experts, further refinements and improvements in the intervention design and components were made to finalise the intervention contents.

The final curriculum of the IATIP is structured around three core components, each tailored to address the nuanced challenges faced by adolescents in Bangladesh. First, the information curriculum aims to cultivate factual awareness by providing students with scientifically grounded insights into the consequences of tobacco use. Second, the social influence curriculum seeks to raise awareness of diverse social influences (e.g., peer pressure) that may encourage tobacco use and learn to manage both direct and indirect high-risk situations with confidence [37]. Complementing this, the third component, the social competence curriculum, focuses on enhancing adolescents’ social learning processes and life skills, such as problem-solving, decision-making, and cognitive skills for resisting various interpersonal or media influences [38]. It aims to strengthen their self-control, self-esteem, coping strategies for stress, and general social and assertive skills, thereby empowering them to refuse offers to tobacco use through improved social competence. While the social competence curriculum fosters broader psychosocial skills, the social influence curriculum specifically addresses the pressures that may lead to tobacco use [37].

### Comparator

Schools in the control arm will continue with their standard curriculum and activities, which include general health education but do not specifically focus on tobacco prevention. These activities typically cover a broad range of health-related topics such as nutrition, physical activity, and hygiene, but do not include targeted interventions aimed at preventing tobacco initiation. To ensure equitable benefit from the research, written materials from the intervention will be shared with control schools after the follow-up assessment.

### Procedure

#### Recruitment

A pool of 24 secondary and higher secondary schools in Dhaka, representing the most common type of school following the National Curriculum and Textbook Board (NCTB) curriculum in Bangla, was invited to participate. This sampling strategy enhances generalizability across the Bangladeshi school system. Half of the invited schools agreed to participate, from which eight schools will be randomly selected. All eligible year 8 and year 9 students within these schools will be included in the study. Invitation letters will be sent to school authorities, and information sheets and consent forms will be distributed to students and their parents or guardians. The final list of participating schools, classes, and students will be confirmed based on eligibility criteria.

#### Randomization

**Fig. 1:**
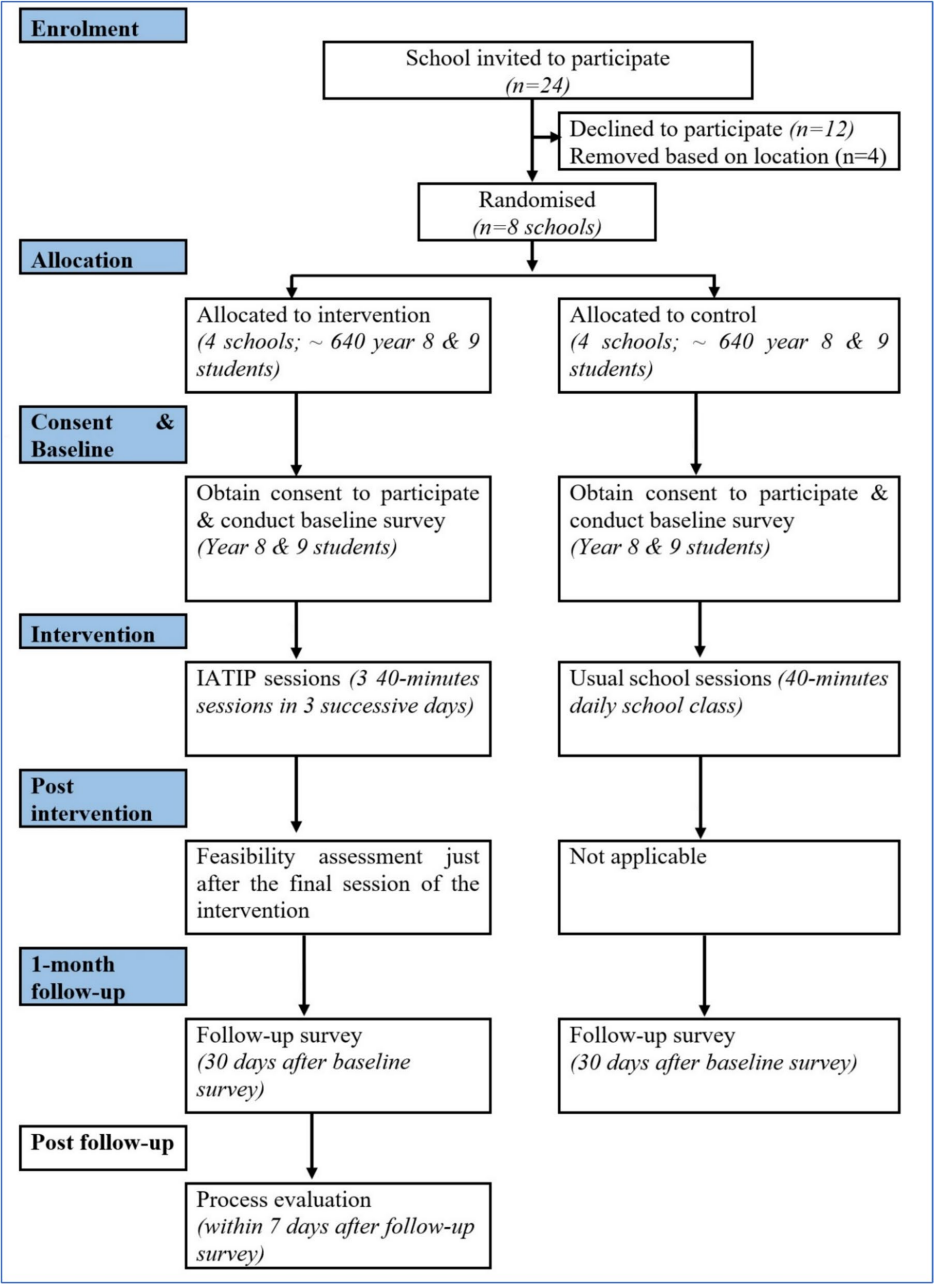
Anticipated recruitment, randomization, and assessment of participants

Given the risk of contamination within schools, individual randomization is impractical. Schools will instead be randomised into intervention or control arms using a cluster design. Stratification and matching will account for school location (based on local government boundaries), monthly tuition fees, and school passing rates to ensure balanced allocation. A random sequence generation method involving three stratified blocks (based on sex composition of the schools: girls only, boys only, and mixed sex) will allocate schools (four in each group) using computer-generated random numbers. Randomization will be concealed until allocation to minimise selection bias. While blinding of participants and researchers is not possible, independent data collectors will collect outcome data to minimise assessment bias. All data will be promptly entered into the UCL Data Safe Haven using REDCap software [39, 40]. The randomization process will be closely monitored, with any deviations from the protocol transparently documented in the final trial report. Figure 1 presents the recruitment and allocation process, while Table 2 outlines the enrolment, interventions, and assessment schedule in the SPIRIT flow diagram.

**Table 2:**
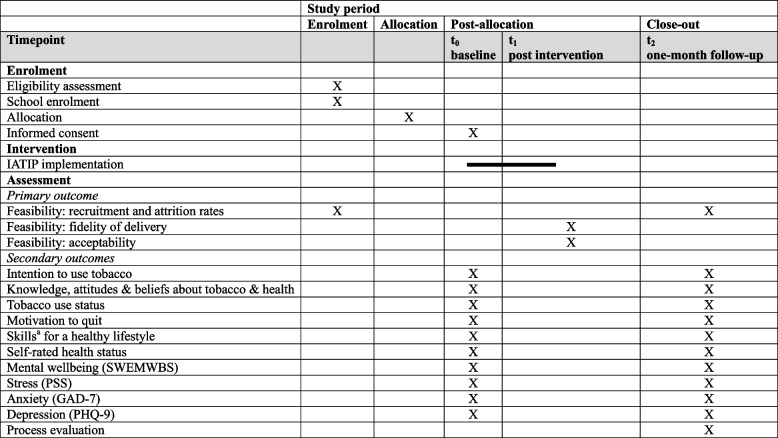
SPIRIT flow diagram: schedule of enrolment, interventions, and assessments

*IATIP* Intervention for Adolescent Tobacco Initiation Prevention, *SWEMWBS* Short Warwick–Edinburgh Mental Well-being Scale, *PSS* Perceived Stress Scale, *GAD-7* Generalized Anxiety Disorder-7 (GAD-7), *PHQ-9* Patient Health Questionnaire-9

^a^ assertiveness, decision-making skill, problem-solving skill, refusal skill, and self-esteem

#### Assessments

The trial will be implemented over a 3-month period, with assessments conducted at three time points: baseline (t_0_), immediate post-intervention for feasibility assessment (t_1_), and at 1-month follow-up (t_2_) (Fig. 1; Table 2). Data collection sessions (30–40 min each) will take place during class time and will involve structured questionnaires administered to both trial arms.

All assessment instruments (questionnaires and interview guides) underwent multiple rounds of expert review by behavioural sciences specialists to ensure clarity and contextual appropriateness. The tools were then translated into Bangla for cultural relevance and accessibility. The initial translation was conducted by a bilingual expert fluent in both English and Bangla, with academic training in public health and experience in health-related research. This was followed by a back-translation into English by two local university graduates whose native language was Bangla and who had a good command of English. A final review by the lead author ensured equivalence across versions. A pilot test was subsequently conducted with a purposive sample of eight adolescents, selected to reflect age and socio-economic diversity. The pilot assessed tool feasibility, clarity, completion time, and overall usability. Based on participant feedback, further refinements were made (e.g., simplifying complex items, clarifying ambiguous terms, and improving formatting) to optimise the instruments for the study population.

## Measures

### Primary outcome measures

#### Feasibility

The primary outcome for this pilot trial is the intervention’s feasibility. Recruitment and attrition rate, fidelity of delivery, and intervention acceptability will be examined. Recruitment success will be assessed by calculating the percentage of eligible participants who agree to participate. Attrition will be monitored by calculating the dropout rate, representing the percentage of recruited participants who do not complete the follow-up assessment. Fidelity of the intervention will be assessed using a pre-defined index, consisting of 21 IATIP content items, evaluated on a 3-point Likert scale (0 = not implemented, 1 = partially implemented, and 2 = fully implemented). Fidelity data will be collected through multiple methods, including video recording observations of intervention delivery as a gold standard [41], self-reports from students [42], and intervention operator delivery logs [43]. In addition, the quality of the sessions delivery by the facilitator will be evaluated on a 5-point Likert scale from 1 (not at all) to 5 (extremely). Participants will be asked, “How would you rate the quality of the sessions’ delivery by facilitator?”.

To evaluate the acceptability of the intervention, participants’ perceptions of the intervention’s relevance and appropriateness will be explored. Participants will be asked a 5-point Likert scale question: “To what extent do you feel the study sessions met your expectations?”, with response options ranging from 1 (not at all) to 5 (completely). They will also be asked, “To what extent do you think the components were relevant to preventing tobacco initiation and promoting health and well-being?” and “To what extent do you think the images and videos presented in the sessions were appropriate and relevant to the contents?” with the same measures. The clarity and comprehensibility of the intervention materials, such as handbooks and presentations, will be rated on a 5-point Likert scale from 1 (not at all) to 5 (extremely). Finally, participants' engagement during the intervention sessions will be assessed using a 5-point Likert scale question: “How engaged did you feel during the taught and interactive sessions?” Response options range from 1 (not at all) to 5 (extremely), with higher scores indicating greater engagement during the sessions.

### Secondary outcome measures

#### Change in intention to initiate tobacco use

To assess the change in intention to initiate tobacco use, a two-item 4-point Likert scale will be used: (1) “if someone (cousin/friend/close relative) offers you a tobacco product, would you use it?” and (2) “at any time during the next 12 months, do you think you will use any form of tobacco?” Responses will range from “Definitely not,” to “Definitely yes”, with scores ranging from 0 to 6. Lower scores indicate a lower likelihood of intending to initiate tobacco. The potential effect of the intervention will be determined by changes in scores between baseline and follow-up.

#### Health and well-being

Health and wellbeing will be assessed using the following validated scales. To assess physical health state, students will rate their overall physical health on a 5-point scale, with higher scores indicating better health. Mental well-being will be measured using the Short Warwick–Edinburgh Mental Well-being Scale, with scores ranging from 7 to 35 [44, 45]. Perceived stress will be evaluated using the 10-item Perceived Stress Scale [46]. This widely recognised scale assesses the extent to which individuals perceive situations in their lives as stressful. Students’ anxiety will be assessed using the Generalized Anxiety Disorder-7 scale (GAD-7), a 7-item 4-point Likert scale, with scores ranging from 0 to 21 [30]. The Patient Health Questionnaire-9 scale (PHQ-9), a 9-item 4-point Likert scale, with scores ranging from 0 to 27, will be used to assess depression [47] These scales, chosen for their validity and reliability, offer a comprehensive evaluation of adolescents' mental health and well-being, encompassing positive aspects, perceived stress, anxiety, and depressive symptoms. The structured responses provided by students will contribute valuable data for understanding the effect of the IATIP on mental health outcomes.

#### Knowledge about tobacco and health

Students’ knowledge about tobacco and health will be assessed using 20 items adapted from a previous study [48]. A student will earn a point for each correct response, with a maximum score of 20.

#### Attitudes toward tobacco and health

Students’ attitudes toward tobacco and health will be measured using a 16-item standardized scale adapted from a previous study conducted in China [48]. Responses will be rated on a 5-point scale, with scores ranging from 16 to 80, and higher scores reflecting more negative attitudes toward tobacco. For positive attitude items, responses of “extremely agree” to “extremely disagree” will be scored as 5 to 1, whereas for negative attitude items, the scoring will be reversed to 1 to 5 points.

#### Beliefs about tobacco and health

Students’ beliefs about tobacco use will be assessed using an 8-item 4-point scale adapted from the EU-Dap questionnaire [49, 50]. This scale focuses on both positive (4 items) and negative (4 items) beliefs concerning various aspects of tobacco use. Positive beliefs include feeling relaxed, having fun, being popular, and being confident and outgoing. Negative beliefs encompass issues with parents and friends, financial problems, and the risk of addiction.

#### Skills for a tobacco-free healthy lifestyle

To comprehensively assess various skills contributing to a tobacco-free healthy lifestyle, multiple dimensions will be evaluated through distinct Likert-type scales. For ‘Decision-Making Skills’, students will respond to statements reflecting different approaches to decision-making, indicating their level of agreement (1 = strongly agree to 4 = strongly disagree) across five items. For ‘Refusal Skills’, students will navigate hypothetical situations, expressing their likelihood of refusal (1 = very likely to 4 = very unlikely) in response to three scenarios. ‘Self-esteem’ will be assessed through agreement with self-descriptive statements (1 = strongly agree to 4 = strongly disagree), encompassing ten items. ‘Poor Problem-Solving Skills’ will be assessed by responses to statements related to interpersonal dealings (1 = strongly agree to 4 = strongly disagree), consisting of five items. Lastly, ‘Assertiveness’ will be evaluated by considering the ease or difficulty students perceive in accomplishing certain tasks (1 = very easy to 4 = very difficult) across six scenarios. This multifaceted approach aims to capture insights into students’ skills, providing a holistic understanding of their capabilities related to maintaining a tobacco-free healthy lifestyle. The scales used for assessing ‘Decision-Making Skills’, ‘Refusal Skills’, ‘Self-Esteem’, ‘Poor Problem-Solving Skills’, and ‘Assertiveness’ were adapted from the EU-Dap questionnaire [49]. The EU-Dap questionnaire is a validated instrument in the field, providing a comprehensive framework for evaluating various skills relevant to tobacco prevention among adolescents.

### Covariates

#### Socio-demographic information

Socio-demographic information will be collected from all participating students through both open-ended and close-ended questions, including their age, sex, religion, weekly pocket money expenditure, place of living (e.g., hostel, rented house with parents, owned property with parents, and others), and family background (including paternal and maternal education levels). See the full questionnaire in the Additional file 1.

#### Tobacco use behaviours

The standard of Global Youth Tobacco Survey (GYTS) will be applied for the calculation of tobacco use prevalence [51]. The estimates of tobacco use will derive from six questions, resulting in three measures: current user, ever user, and non-user. A tobacco user will be defined as someone who is currently using ≥ 1 tobacco product (combustible: cigarettes, pipes, cigars, waterpipes, hookah, shisha, bidis, and non-combustible: tobacco leaf, gul, zorda with pan, khaini, and pan-masala). Current tobacco user includes daily and occasional tobacco user in the past 30 days preceding the data collection. Ever tobacco user will be defined as someone who has consumed ≥ 1 tobacco product during their lifetime. Non-user will be defined as someone who has never used any tobacco products.

#### Cost measures

All direct costs, including intervention delivery costs (based on materials purchased and researcher time), will be recorded. An estimation will then be calculated to determine whether the costs are realistic and feasible based on available funding and resources for the future large-scale definitive trial.

### Data management

To ensure data integrity, confidentiality, and security, strict measures will be implemented, complying with the Data Protection Act 2018 and General Data Protection Regulations. Participant-completed questionnaires will be securely stored after data entry in a locked cupboard in Bangladesh under SH’s control. An anonymous soft copy of the dataset will be stored in a password-protected computer at University College London for 10 years, facilitating future assessments.

### Progression criteria

To inform the decision on whether to proceed to a future definitive trial, pre-specified progression criteria will be applied using a traffic light system [52].

(i) Recruitment: achieving ≥ 80% of the planned school and student recruitment will be considered green (go), 60–79% amber (amend), and < 60% red (stop).
(ii) Retention: follow-up completion rates of ≥ 85% will be considered green, 60–84% amber, and < 60% red.
(iii) Intervention fidelity: delivery of ≥ 85% of the intended sessions with high adherence to protocol will be green, 60–84% amber, and < 60% red.
(iv) Acceptability: if ≥ 80% of adolescents and teachers rate the intervention as acceptable or very acceptable, this will be green, 60–79% amber, and < 60% red.

These thresholds were informed by benchmarks reported in previous pilot or feasibility trials [53–55] and reflect the minimum requirements for a larger trial to be feasible, sufficiently powered, and methodologically robust. Findings will be interpreted in light of these criteria to decide whether the intervention is feasible for a larger definitive trial, whether modifications are required, or whether the trial should not progress.

### Sample size calculations

Formal sample size calculations are not typically required for pilot or feasibility studies [56]. The sample size for this trial was therefore guided primarily by feasibility considerations, particularly the need to estimate key parameters such as recruitment and retention rates, adherence to the intervention, and completeness of outcome data with acceptable precision. For example, with an overall sample of approximately 1280 participants (640 in each group), a retention rate of 85% could be estimated with a 95% confidence interval of ± 2.0%, providing a sufficiently precise estimate to inform planning for a future definitive trial [57, 58].

In addition to feasibility considerations, an exploratory calculation was conducted to provide a pragmatic estimate for recruitment targets and to assess the potential scale of a future definitive trial. This calculation, based on a potential efficacy outcome (intention to initiate tobacco use), is not intended to serve as the primary justification for the sample size. Instead, it offers supportive information by (i) demonstrating the plausibility of detecting an effect of interest, (ii) informing logistical planning and resource allocation, and (iii) providing preliminary data to estimate the variability of outcomes for a full-scale trial. For completeness, the details of this calculation are reported in Additional file 2.

### Analysis

The primary focus of this pilot trial is feasibility. Descriptive analyses will be undertaken to profile baseline characteristics of both the intervention and control groups, and to assess key feasibility outcomes. These include rates of recruitment, consent, retention, and adherence, as well as indicators of intervention acceptability and engagement at both the cluster and individual levels. These feasibility outcomes will be reported with corresponding proportions, means, and 95% confidence intervals, where appropriate. Pre-specified progression criteria (see above) will be applied to determine the feasibility of proceeding to a definitive trial.

The unit of analysis will be the individual adolescent, although randomization occurs at the school level. Clustering by school and class will be accounted for in multilevel models. Exploratory analyses of potential intervention effects will also be conducted. For binary outcomes, binomial distributions will be used, while normal distributions will be applied for continuous variables. Assumptions of normality, homogeneity of variance, and linearity will be examined; if violated, appropriate transformations (e.g., logarithmic or square root) will be applied. For missing data exceeding 5%, patterns and mechanisms of missingness will be explored, with multiple imputation used as appropriate. Sensitivity analyses will be undertaken to examine the impact of missing data and outliers.

To evaluate the potential efficacy of the intervention in reducing intention to use tobacco, a mixed-effects model will be employed. These models will control for baseline covariates at the individual, class, and school levels, incorporate minimization factors, and adjust for clustering by school. Given the small number of clusters (eight schools), small-sample corrections will be applied to reduce the risk of biased inferences. For continuous outcomes, the Kenward–Roger approximation (REML estimation) will be used to adjust standard errors and denominator degrees of freedom; if convergence issues arise, the Satterthwaite approximation will be applied. For binary or other non-Gaussian outcomes, simple cluster-level analyses will be conducted (e.g., calculating school-level outcome proportions and comparing these between groups using t-tests or non-parametric tests). All results will be interpreted with caution in light of the limited number of clusters, focusing on the direction and magnitude of the effect rather than on p-values, or highlighting the uncertainty in the estimates. Analyses will follow an intention-to-treat principle, supplemented by sensitivity analyses using multiple imputation.

Secondary exploratory analyses will adopt a repeated-measures approach to assess changes in tobacco knowledge, attitudes, beliefs, resistance skills, and mental well-being. Subgroup analyses will also be conducted (e.g., school year group = year 8 vs year 9, sex = male *vs* female, housing = living with parents vs without parents, family socioeconomic status = low-income *vs* middle/high-income, family/friends’ tobacco use = yes vs no, mental health condition = good vs poor, etc.) to provide preliminary estimates of potential differential effects. These analyses are exploratory, intended to generate hypotheses and to inform the design of a future definitive trial. Findings will be interpreted with extreme caution and will not be considered confirmatory.

All analyses will be conducted using Stata version 18.0, with the level of significance set at *p* < 0.05 for exploratory outcomes.

### Process evaluation

#### For students

A post-intervention process evaluation will be conducted at the end of the 1-month follow-up among a sub-sample of pupils, using a mixed-methods approach to gain insights into intervention implementation, facilitation, participant experiences, and potential areas for improvement. Participants (16 students) will be purposively sampled for diversity in sex and engagement levels.

Separate from the primary outcome, the process evaluation for students will include measures to understand barriers and facilitators to participation, collect feedback on intervention acceptability, and assess participant engagement and perceived program efficacy. Feedback will be gathered on challenges in attending sessions and assessments. To evaluate the participants’ perceptions of the intervention's acceptability, they will be asked to identify the components they found most helpful or least helpful, providing reasons. Participant engagement will be explored, considering factors that influenced engagement positively or negatively. Perceived program efficacy will be evaluated by assessing beliefs about the intervention's impact on improving knowledge, attitudes, and behaviours related to tobacco, preventing tobacco initiation, enhancing life skills, and promoting health and well-being qualitatively and quantitatively. Participants will be asked to rate their responses on 5-point Likert scales, ranging from 1 (not at all) to 5 (a great deal), for questions such as “To what extent do you think the intervention helped to improve your life skills, such as decision-making and problem-solving?”, “To what extent do you believe that the intervention will help prevent tobacco initiation among school adolescents?”, and “Do you believe the intervention contributed to the promotion of mental well-being among adolescents?” Open-ended questions will also be included, such as in your opinion, how has the study influenced your knowledge, attitudes, and behaviours related to tobacco use? Furthermore, participants will be asked whether the interactive activities were helpful in enhancing their understanding of tobacco prevention and health and well-being promotion. Finally, feedback and suggestions for improvement will be sought to inform future refinements of the IATIP.

#### For teachers

Similarly, a post-intervention process evaluation will be conducted among a sub-sample of teachers, using the same mixed-method approach. Participants (4 teachers) will be purposively sampled for diversity in sex and engagement levels. The process evaluation for teachers will include measures to understand their experiences in facilitating the intervention, challenges faced, and suggestions for improvement. Additionally, their perceptions of the intervention’s acceptability and efficacy will be assessed using similar qualitative and quantitative methods as outlined for the students.

#### Analysis

For process evaluation, both quantitative and qualitative analyses will be conducted. Descriptive analyses will handle quantitative variables, while the Framework Method [59] will be used for the qualitative variables. Initially, all data will be translated from Bangla to English for better management. Transcripts will be read repeatedly to become familiar with the data. Data coding will follow, with initial transcripts coded by multiple individuals to establish a set of codes. These codes will then be grouped into categories to form a working analytical framework. Subsequent transcripts will be indexed using this framework. Summarizing data by category, a spreadsheet will be utilised to chart the data into a matrix. Finally, data will be interpreted.

To ensure the rigor of the qualitative component, several strategies will be employed. Credibility will be enhanced through researcher triangulation, where multiple members of the research team will independently code a subset of transcripts and resolve discrepancies through discussion. A reflexive approach will be adopted to minimise researcher bias, with team members documenting their assumptions and analytic reflections during the coding process. Transferability will be supported through the use of thick descriptions of the study context and participant characteristics.

## Discussion

The IATIP trial represents a significant endeavour to address the challenges of adolescent tobacco initiation and mental well-being in Bangladesh. To the best of our knowledge, this is the first pilot trial focused on both tobacco prevention and the promotion of mental health among school-going adolescents in Dhaka, Bangladesh.

Bangladesh faces a unique set of challenges in curbing adolescent tobacco use. The widespread availability of tobacco products, weak enforcement of existing regulations, and the absence of targeted prevention interventions contribute to high rates of tobacco initiation [24, 25]. The co-occurrence of tobacco use and common mental health problems among adolescents underscores the need for integrated preventive strategies. The IATIP trial responds to these pressing concerns through the introduction of a novel intervention specifically designed for the Bangladeshi context. Drawing on global evidence that emphasises the effectiveness of school-based interventions [21], IATIP utilises the school setting to reach a large proportion of adolescents. By focusing on both tobacco prevention and the promotion of mental health and well-being, the trial aims to address a critical gap in the current public health landscape.

The study design incorporates rigorous methodologies, including randomization and comprehensive data collection, to ensure robust and reliable findings. The potential impact of this pilot trial extends beyond its immediate goals. If successfully implemented, it may reduce adolescents’ intention to use tobacco, minimise exposure to second-hand smoke, and enhance mental well-being. Given the scalability of school-based interventions, this pilot may inform the development of a future large-scale definitive trial to assess long-term impacts on tobacco use. Furthermore, its success could influence national health policies and strategies. The IATIP trial also aligns with global efforts to achieve the Sustainable Development Goals, particularly Goal 3: Good Health and Well-being [14]. By integrating tobacco prevention and mental health promotion into the school curriculum, the trial contributes to a holistic and sustainable approach to adolescent health.

Nevertheless, several challenges and limitations merit consideration. One anticipated challenge is participant attrition. To mitigate this risk, the study will employ retention strategies, including regular communication with schools and participants. In addition, a prize-giving ceremony for a poster competition, scheduled shortly after the follow-up assessment, will be organised to promote participant engagement and retention [60]. Some limitations may arise from the reliance on self-reported data and the relatively short follow-up period. Although self-reporting is widely used and valuable for assessing subjective experiences, it is susceptible to recall bias and social desirability bias. The short follow-up period may not adequately capture long-term behaviours and outcomes related to tobacco use and mental well-being, thereby limiting the generalizability of findings. To address these concerns, standardised questionnaires, clear guidance, and assurances of confidentiality will be used to improve data reliability. The study’s focus on a specific type of school in Dhaka may further limit the broader applicability of the findings to other school settings or regions within Bangladesh. Ensuring uniform delivery of the intervention across schools may also present logistical challenges, due to variation in school timetables, resource availability, and levels of institutional support. Such inconsistencies could affect intervention fidelity. Further challenges may arise in maintaining consistent and meaningful engagement from students, which could be influenced by absenteeism, lack of interest, or competing academic demands. To address this, interactive and engaging activities will be embedded within the intervention, alongside the promotion of a supportive and inclusive school environment. Addressing these limitations will be essential for the accurate interpretation of the study findings and for establishing the feasibility and potential effectiveness of the intervention in the target population.

## Conclusions

The IATIP intervention has been developed through a robust, evidence-based approach and will be evaluated using a methodologically rigorous pilot trial to assess its potential in addressing pressing public health concerns among adolescents in Bangladesh. Should the intervention prove feasible and show promising efficacy, the study findings will carry important implications for the advancement of tobacco prevention and the promotion of adolescent health and well-being in this context. Positive results would provide a strong rationale for progressing to a full-scale definitive trial. In the longer term, successful implementation at scale could support the integration of IATIP into national strategies, thereby reaching a wider population of adolescents and contributing meaningfully to improved public health outcomes in Bangladesh.

### Protocol amendments

All amendments to the current version of the trial protocol (version v1.11 dated on 15 May 2024) will first be discussed with the Lead Investigator and then submitted to the respective research ethics committees for formal approval. A judgment will be made on the nature of the amendment (i.e., major or minor), applying guidance from the ethics committee.

### Dissemination policy

The results of this trial will be published in a PhD thesis, peer-reviewed journal articles, presentations at scientific conferences, and press releases. All study materials and analysis protocols will be made available on the Open Science Framework (https://osf.io/).

## Supplementary Information

Additional file 1. Trial questionnaires and interview guides

Additional file 2. Sample size calculations – a confidence interval approach

Additional file 3: SPIRIT 2013 Checklist: Recommended items to address in a clinical trial protocol and related documents

## Data Availability

Not applicable.

## Abbreviations

ABC: Arena Blended Connected
BCTs: Behaviour Change Techniques
BCW: Behaviour Change Wheel
C-RCT: Cluster Randomied Controlled Trial
GAD: Generalized Anxiety Disorder
GYTS: Global Youth Tobacco Survey
IATIP: Intervention for Adolescent Tobacco Initiation Prevention
ITT: Intention-to-treat
LMICs: Low- and Middle-Income Countries
NCTB: National Curriculum and Textbook Board
PHQ: Patient Health Questionnaire
PSS: Perceived Stress Scale
SWEMWBS: Short Warwick–Edinburgh Mental Well-being Scale
TDF: Theoretical Domains Framework
