# Supplementary material for: Intervention for adolescent tobacco initiation prevention (IATIP) to promote health and well-being: a protocol for a pilot cluster randomised controlled trial in Dhaka, Bangladesh
Source: Pilot Feasibility Stud. 2025 Nov 17;11:145. doi: 10.1186/s40814-025-01710-9 (PMC12625117; doi:10.1186/s40814-025-01710-9)
Supplement: Supplementary file 1 — Additional file 1. Trial questionnaires and interview guides [file 40814_2025_1710_MOESM1_ESM.docx]

**LONDON’S GLOBAL UNIVERSITY**

School of Life and Medical Sciences

Faculty of Population Health Science

Institute of Epidemiology and Health Care

Department of Behavioural Science and Health

Title of study:

**Intervention for Adolescent Tobacco Initiation Prevention to Promote Health and Well-being**

UCL Research Ethics Committee Approval ID Number: 22645/004

JU Biosafety, Biosecurity & Ethical Committee Approval ID Number: BBEC,JU/M 2024/ 02 (82)

**Baseline Questionnaire – v1.5**

**Instructions**

- Please read each question carefully before answering it.
- Choose the answer that best describes what you believe and feel to be correct.
- Some of the questions will ask about your knowledge, attitudes, and beliefs about using tobacco products.
- Other questions may ask about smoking tobacco in general that includes cigarettes and other types of smoked tobacco products (e.g., Pipes, Cigars, Waterpipes/hookah/shisha, Bidis).
- Other questions may ask about using smokeless tobacco (e.g., Snuff, Chewing tobacco–zarda with pan, tobacco leaf, gul, panmasala–Betel quid with tobacco, Gutka), which is tobacco that is not smoked, but is sniffed through the nose, held in the mouth, or chewed.
- Some of the questions will test your skills for a healthy lifestyle.
- Finally, other questions may ask about your mental health and well-being.

**Identification**

| **SL** | **Question** | **Response options** |
| --- | --- | --- |
| ID1 | School code |  |
| ID2 | Student year |  |
| ID3 | Student section |  |
| ID4 | Student class roll number |  |
| ID5 | Initials of the student’s name |  |

**Section A: Demographic information**

The following questions ask for some background information about yourself.

| **SL** | **Question** | **Response options** |
| --- | --- | --- |
| A1 | How old are you now? | ……………………………………..in years |
| A2 | What is your sex? | 0 = Female  1 = Male |
| A3 | What is your religion? | 0 = No religious belief  1 = Islam  2 = Hinduism  3 = Buddhism  4 = Christianity |
| A4 | How many siblings do you have? | …………………… (in number) |
| A5 | What is the highest level of education of your mother? | 0 = No formal education  1 = School level  2 = University level |
| A6 | What is the highest level of education of your Father? | 0 = No formal education  1 = School level  2 = University level |
| A7 | During an average week, how much money do you have that you can spend on yourself, however you want? (in Taka) | ……………………..Taka |
| A8 | What is the current status of your housing tenure/ living accommodation? | 1 = Hostel  2 = Rented house with parents  3 = Owned property with parents  0 = Other (Please specify……………….) |
| A9 | Do any of the people you are connected with smoke cigarettes? (**Tick all that apply**) | 0 = None/Don’t know  1 = Family in your household  2 = Family not in household  3 = Friends  4 = School teachers |
| A10 | Do any of the people you are connected with use smokeless tobacco products? (**Tick all that apply**) | 0 = None/Don’t know  1 = Family in your household  2 = Family not in household  3 = Friends  4 = School teachers |

**Section B: Information about exposure to second-hand tobacco smoke, intention-to use tobacco and tobacco use behaviour.**

The following questions ask about your exposure to other people’s smoking, susceptibility to tobacco and your own tobacco use behaviour.

| **SL** | **Question** | **Response options** |
| --- | --- | --- |
| **Exposure to second-hand tobacco smoke** | | |
| B1 | During the past 7 days, on how many days has anyone smoked inside your home, in your presence? | 0 = 0 days  1 = 1 or 2 days  2 = 3 to 4 days  3 = 5 to 6 days  4 = 7 days |
| B2 | During the past 7 days, on how many days has anyone smoked in your presence, inside any enclosed public place, other than your home (such as: school, shops, restaurants, shopping malls, movie theatres, any office, inside bus, inside train)? | 0 = 0 days  1 = 1 or 2 days  2 = 3 to 4 days  3 = 5 to 6 days  4 = 7 days |
| B3 | During the past 7 days, on how many days has anyone smoked in your presence, at any outdoor public place (such as: playgrounds, sidewalks, entrance to buildings, parks, beaches, bus terminal, railway station)? | 0 = 0 days  1 = 1 or 2 days  2 = 3 to 4 days  3 = 5 to 6 days  4 = 7 days |
| **Intention to initiate tobacco use** | | |
| B4 | If someone (cousin/friend/close relative) offers you a tobacco product, would you use it? | 0 = Definitely not  1 = Probably not  2 = Probably yes  3 = Definitely yes |
| B5 | At any time during the next 12 months, do you think you will use any form of tobacco? | 0 = Definitely not  1 = Probably not  2 = Probably yes  3 = Definitely yes |
| **Tobacco use behaviour** | | |
| B6 | Have you ever tried or experimented with cigarette smoking? | 0 = No  1 = Yes |
| B7 | During the past 30 days, on how many days did you smoke cigarettes? | 0 = 0 days  1 = 1 or 2 days  2 = 3 to 5 days  3 = 6 to 9 days  4 = 10 to 19 days  5 = 20 to 29 days  6 = All 30 days |
| B8 | Have you ever tried or experimented with any form of smoked tobacco products other than cigarettes (pipes, cigars, waterpipes, hookah, shisha, bidis)? | 0 = No  1 = Yes |
| B9 | During the past 30 days, on how many days did you use any form of smoked tobacco products other than cigarettes (e.g., pipes, cigars, waterpipes, hookah, shisha, bidis)? | 0 = 0 days  1 = 1 or 2 days  2 = 3 to 5 days  3 = 6 to 9 days  4 = 10 to 19 days  5 = 20 to 29 days  6 = All 30 days |
| B10 | Have you ever tried or experimented with any form of smokeless tobacco products (zorda with pan, tobacco leaf, gul, khaini, panmasala)? | 0 = No  1 = Yes |
| B11 | During the past 30 days, on how many days did you use smokeless tobacco? | 0 = 0 days  1 = 1 or 2 days  2 = 3 to 5 days  3 = 6 to 9 days  4 = 10 to 19 days  5 = 20 to 29 days  6 = All 30 days |
| B12 | Would you please let us know about your motivation toward stopping tobacco use?  (Please select the best option for you from the list) | 0 = Not applicable  1 = I don't want to stop tobacco use  2 = I think I should stop tobacco use but don't really want to  3 = I want to stop tobacco use but haven't thought about when  4 = I REALLY want to stop tobacco use but I don't know when I will  5 = I want to stop tobacco use and hope soon  6 = I REALLY want to stop tobacco use and intend to in the next 3 months  7 = I REALLY want to stop tobacco use and intend to in the next month |
| B13 | During the past 12 months, have you ever try to stop tobacco use? | 0 = No  1 = Yes  2 = Not applicable |

**Section C: Knowledge, attitudes and beliefs about tobacco and health.**

The following questions ask about your knowledge, attitudes and beliefs about using tobacco.

| **SL** | **Question** | **Response options** |
| --- | --- | --- |
| **Items of Knowledge** | |  |
| ***Harm related*** | |  |
| C1 | How much do you think people harm themselves when they use tobacco? (Absolute harm perception) | 0 = Don’t know  1 = No harm  2 = Very little harm  3 = Some harm  4 = A lot of harm |
| C2 | In your opinion, do you think that smokeless tobacco products are less harmful, about the same, or more harmful to a person's health than smoking tobacco products? (Relative harm perception) | 0 = Don’t know  1 = Less harmful  2 = Same harmful  3 = More harmful |
| C3 | Which of the following are harmful components of tobacco products? (**Tick all that apply**) | 0 = None/Don’t know  1 = Nicotine  2 = Tar  3 = Carbon monoxide  4 = Hydrogen cyanide  5 = Cadmium |
| C4 | Is ‘Nicotine’ the main substance in tobacco that makes people want to use tobacco products? | 0 = Don’t know  1 = Definitely yes  2 = Probably yes  3 = Probably not  4 = Definitely not |
| C5 | Is ‘Nicotine’ the chemical that causes most of the cancer caused by tobacco use? | 0 = Don’t know  1 = Definitely yes  2 = Probably yes  3 = Probably not  4 = Definitely not |
| C6 | Is a pregnant woman’s tobacco use harmful to her baby? | 0 = Don’t know  1 = Definitely yes  2 = Probably yes  3 = Probably not  4 = Definitely not |
| **Disease related** | |  |
| C7 | Which of the following diseases are caused by tobacco use? (**Tick all that apply**) | 0 = None/Don’t know  1 = Cold  2 = Chronic obstructive pulmonary disease  3 = Lung cancer  4 = Diabetes  5 = Heart disease  6 = Hypertension  7 = Stroke  8 = Bladder cancer  9 = Tuberculosis  10 = Skin problems |

**C8. Attitudes Toward Tobacco and Health**

What do you think about the following statements:

|  |  | 1 = Extremely disagree | 2 = Disagree | 3 = Unclear | 4 = Agree | 5 = Extremely agree |
| --- | --- | --- | --- | --- | --- | --- |
| **Effects of tobacco** | | | | | | |
| a | Tobacco can relieve fatigue, stress and improve study efficiency |  |  |  |  |  |
| b | The feeling of tobacco use after meals is just like being a happy spirit |  |  |  |  |  |
| c | All types of tobacco are very harmful to health |  |  |  |  |  |
| d | We should try to increase job positions by developing tobacco industry |  |  |  |  |  |
| **Smoking image** | | | | | | |
| e | A man without smoking is not a real man |  |  |  |  |  |
| f | It is a fashion for young boys and girls to smoke |  |  |  |  |  |
| g | A brave youth should try the feeling of smoking |  |  |  |  |  |
| h | Smoking is a symbol of maturation and independence |  |  |  |  |  |
| **Tobacco’s role in social contact** | | | | | | |
| i | It is impolite to refuse the tobacco passed by others |  |  |  |  |  |
| j | It is easy to get close relationship by passing tobacco each other |  |  |  |  |  |
| k | Parents should not use tobacco in front of children |  |  |  |  |  |
| l | It is uncivil to use tobacco (e.g., cigarette smoking) in public places |  |  |  |  |  |
| **Tobacco control measures** | | | | | | |
| m | Smoking should be banned in public places |  |  |  |  |  |
| n | All the tobacco advertisements should be prohibited |  |  |  |  |  |
| o | Tobacco sales to minors should be banned |  |  |  |  |  |
| p | No one should be allowed to use tobacco in school area |  |  |  |  |  |

**Beliefs about Tobacco and Health**

| **C9.** How likely is that each of the following will happen to you if you use tobacco in the next month? (**Mark the answer that is closest to your opinion**). | | | | | |
| --- | --- | --- | --- | --- | --- |
|  |  | 1=Very Likely | 2=Likely | 3=Unlikely | 4=Very Unlikely |
| a | Get into trouble with parents |  |  |  |  |
| b | Have problems with my friends |  |  |  |  |
| c | Become an addict |  |  |  |  |
| d | Have money problems |  |  |  |  |
| e | Feel more relaxed |  |  |  |  |
| f | Have more fun |  |  |  |  |
| g | Be more popular |  |  |  |  |
| h | Be more confident and outgoing |  |  |  |  |

C10. Once someone has started using tobacco, do you think it would be difficult for them to quit?

0 = Definitely not

1 = Probably not

2 = Probably yes

3 = Definitely yes

**Section D: Skills for a Tobacco Free Healthy Lifestyle**

**D1. Decision making skills**

There are several possible ways to take decisions. How well do the following apply to you? Mark the answer that is closest to your opinion.

|  |  | 1=Strongly Agree | 2=Agree | 3=Disagree | 4=Strongly Disagree |
| --- | --- | --- | --- | --- | --- |
| a | When I have decided to do something, I always carry it through |  |  |  |  |
| b | I often make up my mind without thinking of the consequences |  |  |  |  |
| c | I weigh up all the choices before I decide on something |  |  |  |  |
| d | I often regret something that I had decided |  |  |  |  |
| e | When I decide on something it doesn’t matter what my friends think |  |  |  |  |

**D2. Refusal skills**

Imagine yourself in each of the following situations. Some of them may be very familiar to you, some others less, so that you may feel less secure in answering. It is enough you do your best. Mark the answer that is closest to your opinion.

|  |  | 1=Very Likely | 2=Likely | 3=Unlikely | 4=Very Unlikely |
| --- | --- | --- | --- | --- | --- |
| a | You and your best friend are at a party where you meet new people, and you feel you really want to get to know them. Someone offers you to use tobacco (e.g., cigarette smoking) together. Your friend accepts. Do you? |  |  |  |  |
| b | You and the same friend are studying hard for an important test at school the day after. Both of you feel stressed and need to calm down. Your friend suggests a cigarette would help, and offers one. Do you accept? |  |  |  |  |
| c | The day after, you both pass the test, and feel now it is time to celebrate. Have still some pocket-money left, and the liquor store is nearby. Would you buy some tobacco (e.g., cigarette) to celebrate? |  |  |  |  |

**D3. Self-esteem**

How much do you agree with the following descriptions of yourself? Mark the answer that is closest to your opinion.

|  |  | 1=Strongly Agree | 2=Agree | 3=Disagree | 4=Strongly Disagree |
| --- | --- | --- | --- | --- | --- |
| a | I feel that I have a number of good qualities |  |  |  |  |
| b | I am able to do things as well as most other people |  |  |  |  |
| c | At times I think I am no good at all |  |  |  |  |
| d | Most boys and girls of my age are smarter than I am |  |  |  |  |
| e | I am quite good at sports |  |  |  |  |
| f | I feel very embarrassed when I have to say something in class |  |  |  |  |
| g | My being happy is important to my parents |  |  |  |  |
| h | I worry a lot about silly things |  |  |  |  |
| i | I often feel nervous over nothing at all |  |  |  |  |
| j | I have plenty of interests and hobbies |  |  |  |  |

**D4. Problem solving skills**

Here are some statements about dealing with other people. Mark the answer that is closest to your opinion.

|  |  | 1=Strongly Agree | 2=Agree | 3=Disagree | 4=Strongly Disagree |
| --- | --- | --- | --- | --- | --- |
| a | When someone tries to make you feel small, you should do the same to them |  |  |  |  |
| b | There is point in letting people know you're angry with them |  |  |  |  |
| c | The only way to deal with bullies is to let them know who is in charge |  |  |  |  |
| d | There are always ways of dealing with problems without having to fight |  |  |  |  |
| e | It is much better to 'fly off the handle' than to explain things calmly |  |  |  |  |

**D5. Assertiveness**

Imagine you would like to do the following things. How easy or difficult would you find

it? Mark one box for each line.

|  |  | 1=Very Easy | 2=Easy | 3=Difficult | 4=Very Difficult |
| --- | --- | --- | --- | --- | --- |
| a | Say something nice to a friend |  |  |  |  |
| b | Ask for a favour |  |  |  |  |
| c | Show someone that I like him/her |  |  |  |  |
| d | Say “no” when someone asks me to do something I do not want to |  |  |  |  |
| e | Call for help when I have got problems |  |  |  |  |
| f | Help someone who needs help |  |  |  |  |

**Section E: Information about mental health and wellbeing**

The following questions ask about your mental health and wellbeing.

**E1. How do you describe your general state of health now? Or, In general, would you say your physical health is:**

1 = Very good

2 = Good

3 = Moderate (in between good and poor)

4 = Poor

5 = Very poor

**E2. Short Warwick-Edinburgh Mental Wellbeing Scale (SWEMWBS)**

|  | Below are some statements about your feelings and thoughts over the last 2 weeks | None of the time  (1) | Rarely  (2) | Some of the time  (3) | Often  (4) | All of the time  (5) |
| --- | --- | --- | --- | --- | --- | --- |
| E2.1 | I’ve been feeling optimistic about the future |  |  |  |  |  |
| E.2 | I’ve been feeling useful |  |  |  |  |  |
| E2.3 | I’ve been feeling relaxed |  |  |  |  |  |
| E2.4 | I’ve been dealing with problems well |  |  |  |  |  |
| E2.5 | I’ve been thinking clearly |  |  |  |  |  |
| E2.6 | I’ve been feeling close to other people |  |  |  |  |  |
| E2.7 | I’ve been able to make up my own mind about things |  |  |  |  |  |

**E3. Perceived stress scale (PSS10). The questions in this scale ask you about your feelings and thoughts during the last month.**

|  | In the last month. how often… | Never  (0) | Almost never  (1) | Sometimes  (2) | Fairly often  (3) | Very often  (4) |
| --- | --- | --- | --- | --- | --- | --- |
| E3.1 | have you been upset because of something that happened unexpectedly? |  |  |  |  |  |
| E3.2 | have you felt that you were unable to control the important things in your life? |  |  |  |  |  |
| E3.3 | have you felt nervous and “stressed”? |  |  |  |  |  |
| E3.4 | have you felt confident about your ability to handle your personal problems? |  |  |  |  |  |
| E3.5 | have you felt that things were going your way? |  |  |  |  |  |
| E3.6 | have you found that you could not cope with all the things that you had to do? |  |  |  |  |  |
| E3.7 | have you been able to control irritations in your life? |  |  |  |  |  |
| E3.8 | have you felt that you were on top of things? |  |  |  |  |  |
| E3.9 | have you been angered because of things that were outside of your control? |  |  |  |  |  |
| E3.10 | have you felt difficulties were piling up so high that you could not overcome them? |  |  |  |  |  |

**E4. Anxiety & E5. Depression assessment**

| E4 | Over the last 2 weeks, how often have you been bothered by any of the following problems? | Not at all  (0) | Several days  (1) | More than half the days  (2) | Nearly every day (3) |
| --- | --- | --- | --- | --- | --- |
| E4.1 | Feeling nervous, anxious |  |  |  |  |
| E4.2 | Not being able to sleep or control worrying? |  |  |  |  |
| E4.3 | Worrying too much about different things? |  |  |  |  |
| E4.4 | Trouble relaxing? |  |  |  |  |
| E4.5 | Being so restless that it is hart to sit still? |  |  |  |  |
| E4.6 | Becoming easily annoyed or irritable? |  |  |  |  |
| E4.7 | Feeling afraid as if something awful might happen? |  |  |  |  |
| E5.1 | Little interest or pleasure in doing things? |  |  |  |  |
| E5.2 | Feeling down, depressed, or hopeless? |  |  |  |  |
| E5.3 | Trouble falling or staying asleep, or sleeping too much? |  |  |  |  |
| E5.4 | Feeling tired or having little energy? |  |  |  |  |
| E5.5 | Poor appetite or overeating? |  |  |  |  |
| E5.6 | Feeling bad about yourself - or that you are a failure or have let yourself or your family down? |  |  |  |  |
| E5.7 | Trouble concentrating on things, such as reading the newspaper or watching television? |  |  |  |  |
| E5.8 | Moving or speaking so slowly that other people could have noticed?  Or, the opposite - being so fidgety or restless that you have been moving around a lot more than usual? |  |  |  |  |
| E5.9 | Thoughts that you would be better off dead, or of hurting yourself in some way? |  |  |  |  |

**End the questionnaire.**

**Thank you very much.**
