# Supplementary material for: Intervention for adolescent tobacco initiation prevention (IATIP) to promote health and well-being: a protocol for a pilot cluster randomised controlled trial in Dhaka, Bangladesh
Source: Pilot Feasibility Stud. 2025 Nov 17;11:145. doi: 10.1186/s40814-025-01710-9 (PMC12625117; doi:10.1186/s40814-025-01710-9)
Supplement: Supplementary file 2 — Additional file 2. Sample size calculations – a confidence interval approach [file 40814_2025_1710_MOESM2_ESM.docx]

**Additional file 2: Sample size calculations – a confidence interval approach**

Assuming the one-sided 80% confidence interval approach for analysis [1], the sample size is calculated as follows:

**N =** $\frac{\left[ \left( \boldsymbol{4}\boldsymbol{\sigma}^{\boldsymbol{2}} \right)\left( \boldsymbol{Z}_{\boldsymbol{1-\alpha}}\boldsymbol{+}\boldsymbol{Z}_{\boldsymbol{1-\beta}} \right)^{\boldsymbol{2}} \right]}{\left( \boldsymbol{E} \right)^{\boldsymbol{2}}}$

Here,

N = total sample size for both groups

σ = assumed standard deviation of each treatment response (both treatments assumed equal)

= 4 (literature suggests quite big variability on intention to treat when measured among adolescents)

$Z_{1-\alpha}$ = related to the chosen significance criterion

= 0.84 (if alpha is 0.2)

[Note: We are using a one-sided confidence interval approach based on a feasibility study for alpha (hence 0.2, rather than 0.05)]

$Z_{1-\beta}$ = related to the chosen power, or sensitivity of the experiment

= 0.84 (if power is 80%)

E = minimum detectable difference between treatment means (mean difference)

= 1.53

N = $\frac{\left[ \left( 4{\times4}^{2} \right)\left( 0.84 + 0.84 \right)^{2} \right]}{\left( 1.53 \right)^{2}}$

N = 78 (39 in each group)

*Note:* ‘E’ was calculated as a 24% reduction in the proportion of participants who reported ever using tobacco from a recent systematic review [2]. Previous studies have opted for Likert scales (they give us more power) with most having an upper limit of 7/8 points. Study by Mohammadi et al., 2019 (which was referenced in the systematic review) found a baseline average of 7 [3].

By calculating the design effect, the sample size will be as follows:

Design effect = 1 + (m − 1) ρ

= 1+ (160 – 1) * 0.08

= 13.7

*Note:* The clusters are schools with two classes in year 8 and 9 participating (each class has 40 students) so the average cluster size (m) is 4*40=160, with the intraclass coefficient (ρ) conservatively estimated to be around 0.08 (deliberately chosen as a conservative estimate reflecting the upper range of values from a recent systematic review) [2].

This would give us a total sample size of 1,068 (N*Design effect = 13.7*78).

Furthermore, considering a 15% non-response and/or lost to follow-up rate with the calculated sample size (1068), the final sample size will be 1280 (640 in each group) or four schools per group and eight schools in total (160*8=1280).

Finally, **N = 1280** (640 in each group).

**References**

1. Cocks K, Torgerson DJ. Sample size calculations for pilot randomized trials: a confidence interval approach. J Clin Epidemiol. 2013;66(2):197-201. Epub 20121127. doi: 10.1016/j.jclinepi.2012.09.002. PubMed PMID: 23195919.

2. Hossain S, Tattan-Birch H, Beard E, Shahab L. Evaluating School-based Interventions for Preventing and Reducing Tobacco use among Adolescents in Low- and Middle-income Countries: A Systematic Review and Meta-Analysis. Am J Prev Med. 2025;69(2):107656. Epub 20250514. doi: 10.1016/j.amepre.2025.107656. PubMed PMID: 40379060.

3. Mohammadi M, Ghaleiha A, Rahnama R. Effectiveness of a peer-led behavioral intervention program on tobacco use-related knowledge, attitude, normative beliefs, and intention to smoke among adolescents at Iranian Public High Schools. International Journal of Preventive Medicine. 2019;10(1). doi: <https://doi.org/10.4103/ijpvm.IJPVM_493_17>.
